# Sepsis in end-stage liver disease and acute-on-chronic liver failure: pathophysiology, diagnostic challenges, and pharmacological management

**DOI:** 10.3389/fphar.2026.1775585

**Published:** 2026-05-07

**Authors:** Jacopo Belfiore, Riccardo Taddei, Lorenzo Roberto Suardi

**Affiliations:** 1 Transplant Anesthesia and Intensive Care Unit, University of Pisa (Italy), Pisa, Italy; 2 Infectious Diseases Unit, Department of Clinical and Experimental Medicine, University of Pisa (Italy), Pisa, Italy

**Keywords:** acute-on-chronic liver failure, antimicrobial pharmacotherapy, cirrhosis-associated immune dysfunction, immunomoulation therapy, sepsis

## Abstract

**Background:**

Sepsis represents a leading cause of acute decompensation, acute-on-chronic liver failure (ACLF), and short-term mortality in patients with end-stage liver disease (ESLD). Its clinical course is shaped by the coexistence of profound systemic inflammation, cirrhosis-associated immune dysfunction (CAID), and early multiorgan failure, which together complicate diagnosis, antimicrobial management, and supportive care.

**Methods:**

This narrative review synthesizes current evidence on the epidemiology, immunopathophysiology, microbiology, diagnostic strategies, and therapeutic management of sepsis in patients with ESLD and ACLF, with a specific focus on pharmacological considerations, antimicrobial resistance, biomarker-guided diagnosis, and emerging immunomodulatory and extracorporeal therapies.

**Results:**

Patients with ESLD and ACLF exhibit a dynamic immune phenotype characterized by impaired innate and adaptive immune responses alongside persistent systemic inflammation, predisposing them to severe infections and sepsis. Multidrug-resistant bacterial pathogens and invasive fungal infections are increasingly prevalent and significantly worsen outcomes. Although rapid molecular diagnostics and selected biomarkers improve early pathogen identification and risk stratification, their diagnostic accuracy remains limited by baseline inflammation and hepatic dysfunction. Empirical antimicrobial therapy must balance early broad-spectrum coverage with antimicrobial stewardship, accounting for altered pharmacokinetics and pharmacodynamics. Supportive strategies—including optimized fluid resuscitation, vasopressor therapy, renal replacement techniques, and extracorporeal blood purification—remain central, whereas immune-modulating therapies such as granulocyte colony-stimulating factor, interleukin-1 blockade, and intravenous immunoglobulins are biologically plausible but not yet supported by robust clinical evidence.

**Conclusion:**

Sepsis in ESLD and ACLF is a complex, high-risk condition requiring an integrated, multidisciplinary approach that combines early diagnosis, individualized pharmacological strategies, and tailored organ support. Despite advances in diagnostics and supportive care, outcomes remain poor, underscoring the urgent need for disease-specific clinical trials to refine antimicrobial strategies and evaluate targeted immunomodulatory interventions in this vulnerable population.

## Highlights

A comprehensive narrative literature search was conducted using the PubMed/MEDLINE, Scopus, and Web of Science databases to identify relevant studies on sepsis in end-stage liver disease (ESLD) and acute-on-chronic liver failure (ACLF). The search covered publications from January 2000 to September 2025, with emphasis on the most recent and clinically relevant evidence. Keywords and Medical Subject Headings (MeSHs) included combinations of ““cirrhosis,”,”, ““end-stage liver disease,”,”, ““acute-on-chronic liver failure,”,”, ““sepsis,”,”, ““infection,”,”, ““antimicrobial therapy,”,”, ““pharmacokinetics,”,”, ““antimicrobial resistance,”,”, ““biomarkers,”,”, and ““extracorporeal therapies.”.”. Original studies, systematic reviews, meta-analyses, randomized controlled trials, and major international guidelines published in English were considered. Articles were selected based on their relevance to pathophysiology, diagnostic strategies, and pharmacological or supportive management, with additional key references identified through manual screening of bibliographies and expert appraisal.

## Introduction: epidemiology of end-stage liver disease/Acute-on-chronic liver failure

In patients with advanced cirrhosis, bacterial infections and sepsis represent a central determinant of disease progression and short-term prognosis, acting both as major precipitants of acute decompensation and as primary drivers of multiorgan failure and death (Piano et al., 2021; Tian et al., 2025). Cirrhosis itself constitutes a major and growing global health burden, accounting for a substantial proportion of liver-related morbidity and mortality worldwide, with complications of advanced disease (including infections) being the leading causes of hospitalization and death (Devarbhavi et al., 2023). Contemporary cohorts consistently demonstrate that approximately 30%–40% of patients admitted with acutely decompensated cirrhosis have an ongoing bacterial infection, which represents the most frequent precipitating event of acute decompensation and acute-on-chronic liver failure (ACLF) (Tian et al., 2025; European Association for the Study of the Liver, 2023). According to the EASL-CLIF definition, ACLF occurs in roughly 25%–35% of hospitalized patients with decompensated cirrhosis and is characterized by intense systemic inflammation, rapid development of extrahepatic organ failures, and extremely high short-term mortality (European Association for the Study of the Liver, 2023). In this setting, sepsis (most commonly arising from spontaneous bacterial peritonitis, pneumonia, and urinary tract infections) constitutes the predominant trigger of ACLF and is associated with higher ACLF grades, greater organ failure burden, and worse outcomes (European Association for the Study of the Liver, 2023; Fernández et al., 2018). The presence of infection in cirrhotic patients is associated with an approximately fourfold increase in mortality compared with non-infected counterparts, with 28- and 90-day mortality exceeding 50%–60% in patients with ACLF grade 2–3 (Fernández et al., 2018; Allhoff et al., 2025). Furthermore, recent European transplant and ICU cohorts highlight the increasing epidemiological relevance of multidrug-resistant organisms (MDROs), which frequently precipitate ACLF or complicate its clinical course and independently predict poor short-term outcomes and post-transplant mortality (Belli et al., 2021). Collectively, these data underscore that sepsis in end-stage liver disease (ESLD) and ACLF is not a coincidental complication but rather a core pathobiological event at the intersection of immune dysfunction, systemic inflammation, and organ failure; it provides the essential epidemiological framework for contemporary discussion of early recognition and management strategies in this uniquely vulnerable population (Devarbhavi et al., 2023; Belli et al., 2021).

## Pathogenesis of cirrhosis-associated immune dysfunction

**FIGURE 1:**
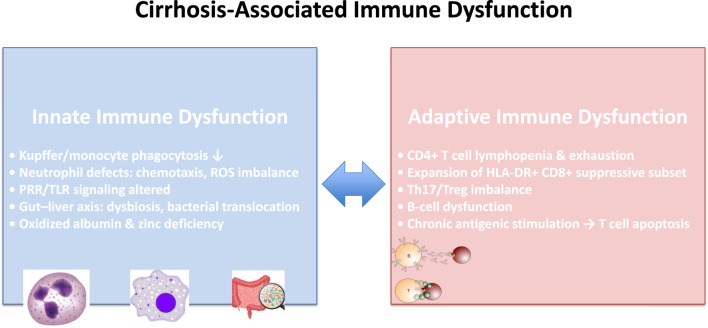
Schematic representation of innate and adaptive immune alterations in CAID with simple icons for cells and microbes. CAID, cirrhosis-associated immune dysfunction; ROS, reactive oxygen species; PRRs, pattern recognition receptors; TLRs, toll-like receptors.

Cirrhosis is increasingly recognized as a systemic disease that extends beyond hepatic structural damage, with profound effects on immune homeostasis. The concept of cirrhosis-associated immune dysfunction (CAID) encompasses a dynamic and bidirectional spectrum of immune abnormalities, ranging from chronic systemic inflammation to profound immunodeficiency, both of which predispose to infectious complications, organ dysfunction, and adverse outcomes (Albillos et al., 2014; Sipeki et al., 2014; Noor and Manoria, 2017). CAID is not a static condition but evolves with disease progression, shifting from a predominantly pro-inflammatory phenotype in compensated or stable decompensated cirrhosis toward immune paralysis in advanced decompensation and ACLF (Albillos et al., 2014; Noor and Manoria, 2017; Irvine et al., 2019) (Figure 1).

### Alterations of innate immunity

Innate immune cells, particularly monocytes, macrophages, neutrophils, and natural killer (NK) cells, are central effectors and targets of CAID. Cirrhosis impairs hepatic immune surveillance by damaging the reticuloendothelial system, reducing the phagocytic capacity of Kupffer cells, and decreasing hepatic synthesis of soluble pattern recognition molecules, such as complement components and lipopolysaccharide-binding protein (Albillos et al., 2014; Sipeki et al., 2014). Neutrophils exhibit both quantitative and qualitative defects, including impaired chemotaxis, phagocytosis, and oxidative burst, and yet paradoxically maintain a propensity for the exaggerated release of reactive oxygen species (ROS) that sustain tissue injury and systemic inflammation (Irvine et al., 2019; Liaskou and Hirschfield, 2019; Wilde and Katsounas, 2019). Monocytes and macrophages show reduced antigen presentation, downregulated HLA-DR expression, and altered cytokine responses, leading to increased vulnerability to bacterial and fungal infections (Noor and Manoria, 2017; Wilde and Katsounas, 2019).

The gut–liver axis plays a pivotal role in perpetuating innate immune dysfunction. Dysbiosis, intestinal barrier disruption, and bacterial translocation result in continuous exposure to pathogen-associated molecular patterns (PAMPs) and damage-associated molecular patterns (DAMPs), which drive systemic inflammation through toll-like receptor (TLR) signaling (Guan et al., 2022; Hasa et al., 2022). Persistent stimulation causes immune cell exhaustion, loss of TLR tolerance, and increased risk of sepsis and multi-organ failure (Wilde and Katsounas, 2019; Guan et al., 2022; Hasa et al., 2022; Engelmann et al., 2023). Moreover, nutritional and metabolic factors contribute to innate dysfunction: zinc deficiency, which is highly prevalent in cirrhosis, impairs neutrophil, NK cell, and macrophage function (Grüngreiff et al., 2016), while oxidized albumin not only loses its antioxidant and immunomodulatory properties but may also generate neoepitopes that sustain systemic inflammation (Wilde and Katsounas, 2019).

### Alterations of adaptive immunity

Although innate immune dysfunction is well established, recent evidence highlights profound defects in adaptive immunity as an equally critical feature of CAID (Sipeki et al., 2014; Noor and Manoria, 2017; Irvine et al., 2019; Liaskou and Hirschfield, 2019). T lymphocytes are quantitatively reduced and functionally impaired, with CD4^+^ T-cell lymphopenia and reduced proliferative responses frequently reported (Wilde and Katsounas, 2019). A key recent finding is the expansion of dysfunctional CD8^+^ T-cell subsets expressing HLA-DR, which exhibit immunosuppressive properties and correlate with poor outcomes (Liaskou and Hirschfield, 2019). Regulatory T cells (Tregs) are often increased, contributing to impaired pathogen clearance, whereas Th17/Treg imbalance has been implicated in systemic inflammation and tissue injury (Hasa et al., 2022). B-cell dysfunction and reduced antibody responses further explain the poor immunogenicity of vaccines in cirrhotic patients (Albillos et al., 2014; Noor and Manoria, 2017).

These adaptive abnormalities are tightly linked to persistent antigenic stimulation from gut-derived bacterial products and hepatocyte injury, promoting chronic activation, exhaustion, and lymphocyte apoptosis (Sipeki et al., 2014; Noor and Manoria, 2017; Irvine et al., 2019; Liaskou and Hirschfield, 2019; Wilde and Katsounas, 2019; Guan et al., 2022; Hasa et al., 2022). The adaptive immune impairment, together with innate cell dysfunction, constitutes the immunodeficient state typical of advanced cirrhosis and ACLF, where susceptibility to life-threatening infections is highest (Engelmann et al., 2023).

### Dynamic spectrum and clinical implications

CAID represents a continuum, where systemic inflammation and immune suppression coexist but predominate at different disease stages. In compensated or early decompensated cirrhosis, systemic inflammation is dominant, driven by DAMPs and PAMPs, and contributes to endothelial dysfunction, portal hypertension, and extrahepatic organ impairment (Albillos et al., 2014; Sipeki et al., 2014; Noor and Manoria, 2017; Irvine et al., 2019; Liaskou and Hirschfield, 2019; Wilde and Katsounas, 2019; Guan et al., 2022; Hasa et al., 2022). As cirrhosis advances, chronic overstimulation leads to immune exhaustion, reduced cytokine production, and impaired pathogen clearance, culminating in immune paralysis during ACLF (Noor and Manoria, 2017; Irvine et al., 2019; Engelmann et al., 2023). This immunological trajectory is clinically relevant: infections occur in up to 35% of hospitalized cirrhotic patients and account for nearly half of all deaths in this population (Irvine et al., 2019; Liaskou and Hirschfield, 2019; Wilde and Katsounas, 2019). Spontaneous bacterial peritonitis remains the most characteristic infection, but urinary tract infections, pneumonia, and bloodstream infections are also common and are frequently associated with multidrug-resistant organisms (MDRO) (Liaskou and Hirschfield, 2019; Hasa et al., 2022).

Understanding the dual nature of CAID is crucial for patient management. The recognition of immune dysfunction as a major pathophysiological hallmark of cirrhosis underscores the need for novel therapeutic strategies, including immunonutrition (e.g., zinc and albumin supplementation), the modulation of gut microbiota, and emerging immune-targeted therapies (Wilde and Katsounas, 2019; Guan et al., 2022; Grüngreiff et al., 2016). Furthermore, biomarkers such as neutrophil-to-lymphocyte ratio, HLA-DR expression on monocytes, and levels of pro-inflammatory cytokines may assist in risk stratification and the early identification of patients prone to sepsis and ACLF (Hasa et al., 2022; Engelmann et al., 2023).

## Epidemiology of bacterial and fungal infections in end-stage liver disease

Infections, both bacterial and fungal, represent high burden diseases in patients affected by so-called ESLD in terms of morbidity, mortality, and healthcare-system-associated costs (Fernández et al., 2012). Several multinational cohorts of patients have reported that up to 50% of infections are community-acquired, while healthcare-associated and nosocomial infections developed in 30%–50% of cases (Piano et al., 2019; Merli et al., 2010; Bartoletti et al., 2014). Interestingly, invasive fungal infections account for less than 10% of culture positive infections and are mainly healthcare/nosocomial infections (Alexopoulou et al., 2015; Verma et al., 2022). Spontaneous bacterial peritonitis (SBP) is the most frequent scenario, accounting for 30%–40% of all infections; it is associated with ACLF and has a high risk of short-term mortality (Jalan et al., 2014). Enterobacterales, Streptococci, and Enterococci are the main pathogens involved in SBP. Following SBP, urinary tract infections (UTIs), pneumonia, bloodstream infections (BSI), catheter-related infections (CLABSIs), and skin and soft tissue infections are commonly reported. The most frequently identified pathogens are Enterobacterales and Enterococci in UTIs and Enterobacterales and Staphylococci in BSI. *Clostridioides difficile* infections, among less frequent infections, has been increasing in cirrhotic patients as secondary infection after broad spectrum antibiotic exposure and multifactorial disruption of intestinal microbiota. In the last 25 years, antimicrobial resistance (AMR) is evolving worldwide and is shaping different epidemiological scenarios between countries and continents. Knowledge of MDROs and hospital “ecology” is fundamental in order to provide the best approach to this difficult-to-treat infections. Some researchers focusing on cirrhotic patients provide prevalence data showing a heterogeneous global AMR scenario where, on the one hand, MDRO are increasing globally over the last 10 years while, on the other hand, specific and unique epidemiological center-to-center or country-to-country AMR patterns need to be considered by the involved physicians (Fernández et al., 2012). Focusing on the main MDROs and their mechanisms, Enterobacterales (such as *E. coli* and *K. pneumoniae*) derive their AMR mainly from enzymatic b-lactams hydrolysis due to the production of b-lactamases (Bush, 2018). These enzymes are capable of destroying the b-lactam ring and making noneffective beta-lactams. Over the last 80 years, beta-lactamases have evolved due to antibiotic pressure and the global spread of MDRO clones (sequencing types-ST) that harbor multimodal AMR features (including not only beta-lactamases but also drug efflux pumps or porin deficit strategy). The most important, and in some countries the most prevalent, among MDRO infections, are Enterobacterales-AMR patterns, including the so-called extended-spectrum beta-lactamases (ESBLs), where CTX-M enzymes (above all CTX-M 15–17) are the most commonly identified, followed by carbapenem-resistant patterns (CRE), in which carbapenemases such as KPC-enzyme and metallo-enzymes (such as NDM, VIM, and IMP) are differently distributed worldwide but are globally increasing. ESBL- and carbapenemase-producing bacteria are resistant to penicillin, cephalosporins, older beta-lactam/beta-lactam inhibitor combinations, and carbapenems. Following the Ambler beta-lactamases classification, ESBL enzymes (CTX-M) and KPC correspond to class A, while metallo-enzymes belong to class B. Class D (with the most important enzyme OXA-48) is similar to class A in terms of AMR, with a different degree of carbapenem hydrolysis ability. Class C identifies the peculiar AmpC, cephalosporinases whose production is constitutive/inducible among *Citrobacter*, *Enterobacter*, and *Serratia*. *Pseudomonas aeruginosa*, another important non-fermenting-gram negative bacteria, responsible for nosocomial infections (above all in ICU settings) such as *Acinetobacter baumannii* and *Stenotrophomonas maltophilia*, rely for AMR on exhibiting intrinsic chromosomal and acquired mechanisms of resistance that are both based on beta-lactamases production (ESBL or carbapenemases or AmpC) and not-enzymes strategies such as drug efflux pumps and porin modifications. The most important Gram positives are *S. aureus* methicillin-resistant (due to MecA gene acquisition) and vancomycin-resistant *Enterococcus faecium* (due to VanA–VanB acquisition) (Sendra et al., 2024). These pathogens exhibit multidrug resistance patterns including resistance to other classes of antibiotics such as aminoglycosides, quinolones, and increasingly, daptomycin and linezolid (once last-chance drugs). Among invasive fungal infections (IFI), candidiasis/candidemia is the most frequent, followed by invasive aspergillosis (Bartoletti et al., 2018). Well known risk factors for IFI are often present in patients with ESLD such as neutropenia, central-venous catheter use with total parenteral nutrition, malnutrition, steroid use, empirical broad spectrum antibiotic exposure, multisite colonization, renal replacement therapy, and prolonged ICU stays. In the last 5 years, fluconazole-resistant *Candida parapsilosis* has increasingly been reported as a critical pathogen spreading worldwide, with the ability to cause persistent nosocomial outbreak and is intrinsically difficult to treat due to its ability to produce biofilm and device-associated infections (Verma et al., 2022; Vena et al., 2025).

### Diagnostic tools

ESLD patients are at heightened risk of severe infections, including those caused by MDRO. Initial work includes clinical, imaging, and microbiological assessments (Piano et al., 2024). Diagnostic paracentesis with ascitic fluid culture and polymorphonuclear count (>250 cells/microl) are mandatory in order to confirm/exclude peritoneal sources of infection and should not be delayed (Orman et al., 2014). Blood cultures, which had been withdrawn due to best practice in order to avoid false positive results due to contamination during the procedure, must be taken before commencing empirical antimicrobial therapy (central venous catheter sampling always simultaneous with peripheral sampling, with at least two sets of both). Urine sediment and culture and sputum culture are useful specimens to investigate in the early phase of sepsis detection, as well a chest X-rays (Karvellas et al., 2024). Rectal swabs for carbapenem-resistant Enterobacteriaceae (CRE) screening utilize molecular assays, most commonly PCR-based, to detect carbapenemase genes (e.g., KPC, NDM, VIM, and IMP) directly from rectal samples. These methods offer rapid turnaround and high sensitivity, facilitating the early identification and isolation of colonized patients, which is critical for infection control and timely effective therapy. The Infectious Diseases Society of America (IDSA) and the American Society for Microbiology (ASM) recommend molecular methods to rapidly detect resistance determinants in clinical microbiology laboratories (Miller et al., 2024). Rapid diagnostics on blood cultures, such as the FilmArray system, employ multiplex PCR panels to identify a broad spectrum of pathogens and resistance genes directly from positive blood cultures (Banerjee and Humphries, 2021). These platforms can reduce time for organism identification and resistance profiling from days to hours, thus enabling earlier targeted antimicrobial therapy. Studies have shown that the implementation of rapid molecular diagnostics in bloodstream infections is associated with more timely therapy and reduced mortality, especially when integrated with antimicrobial stewardship programs (Hattab et al., 2024). The clinical impact is most pronounced in patients with sepsis or MDRO bacteremia, where delays in appropriate therapy are associated with poor outcomes (Sambri et al., 2025; Timbrook et al., 2017). Multiplex PCR panels on bronchoalveolar lavage (BAL) samples are increasingly used to diagnose respiratory infections in immunocompromised hosts, including those with cirrhosis. These panels can simultaneously detect bacterial, viral, and fungal pathogens, as well as resistance genes, with higher sensitivity than conventional cultures, particularly for fastidious or non-cultivable organisms. PCR-based assays and metagenomic next-generation sequencing (mNGS) have demonstrated superior pathogen detection rates and can identify mixed infections, which are common in this population (Li and Liu, 2025). However, interpretation requires clinical correlation due to the potential detection of colonizing or non-viable organisms. The IDSA and ASM recommend BAL sampling and molecular testing as part of a comprehensive diagnostic algorithm for pneumonia in immunocompromised patients. In summary, molecular diagnostic approaches—rectal CRE screening, rapid blood culture PCR, and multiplex BAL PCR—offer significant advantages in the sensitivity, speed, and breadth of pathogen detection in ESLD patients (Miller et al., 2024). Their integration into clinical workflows supports early, optimized antimicrobial therapy and improved infection control.

### Biomarkers

Although pathogen detection in biological samples is widely accepted as the most reliable method of diagnosing infections, clinical microbiological culture and identification processes are slow, with turnaround times of 48–72 h or even more. This underscores the critical need to develop early sepsis diagnostic markers. An ideal biomarker should be cost-effective, non-invasive, highly sensitive, and specific, rapidly available to serve both as a diagnostic tool and monitoring parameter for the point-of-care assessment of sepsis progression and treatment effectiveness (He et al., 2024). Early sepsis is characterized by a increase in inflammatory and endothelial biomarker levels from their baseline. These baseline levels are higher in patients with end-stage liver disease than the general population (Dasgupta et al., 2025). Likewise, ACLF triggers a marked systemic inflammation driven by tissue damage, which mimics a dysregulated host response to pathogens. Thus, differentiating sterile systemic inflammation from sepsis becomes more challenging in these populations (Kumar et al., 2025). Fever is not a reliable sign and can be absent in up to 56% of patients with ACLF (Shalimar et al., 2017; Chen et al., 2021). Both total leucocyte counts and neutrophil-lymphocyte ratios (NLRs) can be elevated in inflammatory states without infection, making them unreliable for discrimination. Although multiple biomarkers for the early diagnosis of sepsis exist, only a few have been studied in relation to liver diseases (Kumar et al., 2025). C-reactive protein (CRP) is an acute-phase protein synthesized in the liver after an inflammatory insult; it therefore lacks specificity for bacterial infections, and its production and metabolism may be altered in patients with liver diseases. In a meta-analysis of CRP accuracy for sepsis in the general population, Tan et al. (2019) showed a pooled sensitivity of 80% but a pooled specificity of only 61%. Furthermore, a lag time of 12–24 h before CRP serum increase has been reported, limiting its usefulness as an early biomarker of sepsis (Kumar et al., 2025). Healthy subjects produce procalcitonin (PCT) in the medullary C-cells of the thyroid gland. Bacterial infections cause an upregulation of the production of the biomarker from different cell types, and so, its measurement has been suggested and validated as a tool for distinguishing bacterial infections from other inflammatory conditions, showing an area under the receiver operating characteristics (AUROC) curve of 85% (Assicot et al., 1993; Wacker et al., 2013). Although PCT appears to be a good tool for detecting bacterial infections in the general population, its discriminatory value in ACLF subjects has been demonstrated to be more limited, with an AUROC curve of 69% (Chen et al., 2021). An infection score comprising serum PCT, CRP, and neutrophil proportion (%) has been evaluated to diagnose bacterial infections in ACLF patients, but the discriminating potential of the score remained modest, with an AUROC curve of 74% (Lin et al., 2020). Interleukin (IL)-6, a pro-inflammatory cytokine, has shown good diagnostic value for differentiating bacterial infections in patients with end-stage liver disease, with a pooled sensitivity and specificity of 85% and 91%, respectively (Wu et al., 2016). Moreover, it has been identified as an independent predictor of mortality in patients with HBV-related ACLF (Kumar et al., 2025; Zhou et al., 2020). Both soluble triggering receptor expressed on myeloid cell-1 (sTREM-1), a receptor implied in the upregulation of the production of pro-inflammatory cytokines, and presepsin, a soluble CD14 subtype, have shown good sensitivity (83%–85%) and moderate specificity (78%–79%) in differentiating sepsis from inflammatory conditions in the general population (Zhang et al., 2015; Qin et al., 2021). In Chen et al. (2021), the diagnostic efficiency of sTREM-1 and presepsin was higher than that of WBC, PCT, and CRP in sepsis diagnosis among ACLF patients. Moreover, combining sTREM-1 or presepsin with the CLIF-SOFA increased diagnostic efficiency, achieving AUROC curves of 87% and 91%, respectively. In Igna et al. (2022), a presepsin level ≥2300 pg/mL was associated with the early diagnosis of bacterial infections in ACLF patients (Table 1).

**TABLE 1:** Report advice on empirical treatments based on risk factors of the Infectious Diseases Society of America (IDSA), the European Society of Clinical Microbiology and Infectious Diseases, and French (SPILF)–Italian (SIMIT) guidelines.

Gram-negative coverage assessment	Low risk for MDRO
Gram-negative coverage assessment	Ceftriaxone/cefotaxime/ceftazidime/cefepime plus metronidazole or amox–clav/piperacillin–tazobactam
Gram-negative coverage assessment	High risk for MDRO
Gram-negative coverage assessment	For ESBL: meropenem/imipenem or carbapenem-sparing strategy (ceftazidime-avibactam, cefepime–enmetazobactam, ceftolozane–tazobactam) For KPC: meropenem–vaborbactam, imipenem–relebactam, ceftazidime–avibactam, cefiderocol, colistin For MBL: avibactam–aztreonam, ceftazidime–avibactam + aztreonam, cefiderocol, colistin For *Pseudomonas aeruginosa* DTR: cefiderocol, ceftolozane–tazobactam plus second agent (aminoglycoside and fosfomycin), colistin For *Acinetobacter* baumannii carbapenem-resistant: cefiderocol plus second agent (ampicillin/sulbactam, tigecycline) or colistin plus second agent, where available durlobactam/sulbactam Warning: Only amox–clav/piperacillin–sulbactam, imipenem and ampicillin/sulbactam have activity on *Enterococcus* spp. if ampicillin-sensitive
Gram-positive coverage assessment (specifically S.aureus methicillin-resistant)	Vancomycin, daptomycin, linezolid, ceftobiprole, ceftaroline, eravacycline
Fungal coverage assessment	For *Candida*: echinocandin or b-liposomal amphotericin For aspergillus/molds: voriconazole, isavuconazole, b-liposomal amphotericin For pneumocystis: co-trimoxazole

MDRO, multi-drug resistant organisms; ESBL, extended-spectrum beta-lactamase; KPC, *Klebsiella pneumoniae* carbapenemase; MBL, metallo-β-lactamase.

### Empirical antimicrobial treatments

According to the Surviving Sepsis Campaign, the administration of antimicrobial therapy is recommended within 1 h of recognition in cases of septic shock or high likelihood for sepsis; in cases of sepsis without shock, it is possible to delay this until 3 h from recognition to allow appropriate investigation (Evans et al., 2021). This clinical assessment is fundamental for a prompt response to critical scenarios but also to avoid unnecessary broad-spectrum antibiotics that could select further antibiotic resistance. A well-based diagnostic momentum will increase the probability of identifying causative pathogens and narrowing the antibiotic treatment to reduce the likelihood of selecting resistant strains and secondary infections (including *C. difficile* infections and candidemia). This approach is supported in nosocomial infections for patients with suspected sepsis referring to emergency departments where the majority of cirrhotic patients are evaluated initially (Nauclér et al., 2021). An appropriate antimicrobial treatment must cover all potential pathogens in case of sepsis or septic shock after quick and proper culture sampling. Abdominal septic source is the first cause of infection, but pneumonia, above all in an ICU setting in immunocompromised patients (Martin-Loeches et al., 2025), and urinary tract infections must be explored in order to provide adequate antibiotic treatment in terms of pharmacokinetic/pharmacodynamic (PK/PD) target and efficacy (Bartoletti et al., 2018; Arabi et al., 2012; Sartelli et al., 2024). In the case of an eventually unknown source of infection, without deferring treatment in case of sepsis/septic shock, the patient will need to undergo imaging studies (e.g., CT scan/echocardiography) to identify the infective focus and exclude mimicking condition (e.g., bleeding and thrombosis). Jointly with the clinical scenario assessment, the physician must be aware of the colonization status of the patient (rectal, nasal, and multisite) by MDRO such as Enterobacterales carbapenemases or ESBL-producing, non-fermenting Gram-negative (*A. baumannii* carbapenem-resistant and *P. aeruginosa*), VRE, MRSA, *Candida albicans*, or other species of this genus. A positive MDRO colonization status must include active antibiotics within the empirical antimicrobial regimen in all cases of suspicion of sepsis or definite septic shock. Clinicians will note the risk for specific MDRO coverage on the individual patient clinical status, suspected source of infection, previous antibiotic treatment, and frailty. The presence of a central venous catheter still remains a principal risk factor both for staphylococcal bacteremia (*S. aureus* or other) and for candidemia. A community-acquired onset of infection (<48 h since admission) should not exclude covering MDRO in high-endemic settings (Timsit et al., 2025). The efficacy of antimicrobials does not only rely on *in vitro* activity; several factors can influence a good clinical response. The pathophysiology of sepsis affects patients with altered volumes of distribution due to third-space expansion that could affect drug serum concentrations as well hypoalbuminemia, which in cirrhotic patients play a critical role (Bajaj et al., 2021). Suboptimal drug serum levels increase not only the risk of clinical failure but also the emergence of antibiotic resistance (Pea and Viale, 2006). Above all, these factors affect hydrophilic antibiotics such beta-lactams and glycopeptides. Therefore, loading doses and extending to continuous infusions are recommended when aiming to optimize PK/PD parameters (Table 2) (Guilhaumou et al., 2019). Second, acute renal failure in patients without history of chronic renal failure and not on dialytic treatment during a septic episode should not lead clinicians to modify antibiotic dosage on the glomerular filtration ratio until 48–72 h of treatment due to probable sepsis-related ARF. Any modification (in terms of dose reduction) during this phase could lead to suboptimal serum levels (Crass et al., 2019). Serum-level drug monitoring should be performed above all in patients with limited therapeutical options (Gatti et al., 2025). Nosocomial or healthcare-acquired infection treatments must be tailored following up-to-date local epidemiology. Recent EASL practice guidelines summarize general recommendations. Table 3 reports advice on empirical treatments based on risk factors from the Infectious Diseases Society of America (IDSA) (Tamma et al., 2024), the European Society of Clinical Microbiology and Infectious Diseases (Paul et al., 2022), and French (SPILF)–Italian (SIMIT) guidelines (Meschiari et al., 2024). Initial empirical antibiotic treatment needs to be evaluated on daily basis and quickly adjusted to microbiological results towards monotherapy if possible. Combination therapy can be suggested rather than monotherapy in cases of deep-seated *S. aureus* infection or for the initial phase in order to achieve blood culture sterilization before embolization or endocarditis onset or for difficult-to-treat non-fermenting Gram-negative (*A. baumannii* carbapenem-resistant, *P. aeruginosa* DTR, *Stenotrophomonas maltophilia*) or specific pathogens and diseases (for severe pulmonary aspergillosis: triazole + b liposomal amphotericin). The literature provides consistent data that most common infections (excluding specific scenarios such as *S. aureus* bacteremia, candidemia, or high inoculum infections) can be successfully treated with short course regimens (The BALANCE Investigators, 2024).

**TABLE 2:** PK/PD implications of end-stage liver disease/Acute-on-chronic liver failure.

Alteration	Effect	Correction
Decreased CYP450 activity (especially CYP3A4 and CYP2E1)	Augmented antibiotic exposure and toxicity	Dosage reduction following childbirth (e.g., tigecycline)
Hypoalbuminemia	Altered antibiotic exposure	Beta-lactams continuous infusion and therapeutic drug monitoring
Renal impairment	Augmented antibiotic exposure and toxicity	Therapeutic drug monitoring
Myelosuppression susceptibility	Anticipated side effect by betalactams or linezolid (cytopenia)	Narrower blood exam monitoring

**TABLE 3:** Summary of the main biomarkers of sepsis in patients with liver failure.

Biomarker	Cut-off	Source	Advantage	Limitation
CRP	25 ng/mL >10 ng/mL for infection	Liver	Wide availability. Low cost	Low specificity for BI. False low levels in liver failure. Lag time: 12–24 h
PCT	0.5 ng/mL	Thyroid	Wide availability. Validated across multiple studies. Rapid elevation, 3–4 h after BI	Modest to poor discriminatory role in liver failure patients. Poor performance in renal failure and immunocompromised patients
IL-6	35 pg/mL	Leucocytes, fibroblasts, monocytes, macrophages, T-cells, endothelial cells	Rapid elevation, 2–4 h after BI	Short half-life. Is a non-specific pro-inflammatory cytokine. Needs further research in liver failure patients
Presepsin	600 pg/mL	Cleavage of fraction of CD-14 receptor	Better performance in liver failure than CRP and PCT. Early detection, <2 h after BI	Limited availability. Expensive. Requires further validation studies
sTREM-1	230 pg/mL	Neutrophils, monocytes, macrophages	Better performance in liver failure than CRP and PCT. Early detection, <2 h after BI. Short half-life (12 min), making it useful for treatment response	Limited availability. Requires further validation studies

CRP, C-reactive protein; BI, bacterial infection; PCT, procalcitonin; IL, interleukin; sTREM-1, soluble triggering receptor expressed on myeloid cell-1.

### Hemodynamic support and organ support strategies

Sepsis represents a precipitating event in patients with ESLD and ACLF and is strongly associated with the development of multi-organ failure and short-term mortality rates, which exceed 30%–50% in advanced stages (European Association for the Study of the Liver, 2023). Cirrhotic patients have baseline systemic vasodilatation, reduced effective arterial blood volume, and often relative or absolute hypo-albuminemia; they can, therefore, present with lower baseline arterial pressure and impaired cardiovascular reserve and autoregulation. Superimposed sepsis frequently causes profound vasoplegia and capillary leak, which worsens tissue hypoperfusion. These features alter typical responses to fluids and vasopressors: aggressive crystalloid administration could lead to over-resuscitation, thus worsening ascites and edema. Vasopressors are often required earlier and at higher cumulative doses to achieve perfusion goals (European Association for the Study of the Liver, 2023; Evans et al., 2021).

#### Fluid management

Fluid administration represents a key step in the hemodynamic management of sepsis, with the aim of preventing tissue hypoxia and preserving organ function. The goal of fluid resuscitation is the early restoration of perfusion to prevent and limit end-organ dysfunction. Currently, the most accepted fluid-resuscitation strategy for early sepsis management is a combination of the dynamic parameters of fluid responsiveness and physiological parameters such as lactate clearance and capillary refill time (Evans et al., 2021). These parameters are often abnormal at baseline in patients with cirrhosis and ACLF, and the optimal endpoints for resuscitation in this population have not yet been determined (Simonetto et al., 2019). Point-of-care ultrasound (POCUS) evaluation provides additional information on the fluid and cardiac status of the patient (cardiac and inferior vena cava (IVC) preload assessment, evaluation for hypovolemic vs. vasodilatory vs. cardiogenic shock, left ventricular and right ventricular function) and may help guide the management and monitoring of hemodynamic and circulatory status (Karvellas et al., 2024; Weiss et al., 2018). The monitoring of dynamic changes in stroke volume, stroke volume variation, pulse pressure variation, or POCUS with fluid challenges or passive leg raise may help guide resuscitation (Karvellas et al., 2024; Premkumar et al., 2021). In patients with large volume ascites, respiratory variation of the IVC could be altered because of the increased abdominal pressure, with the IVC diameter sometimes appearing falsely reduced and its collapsibility abolished (Via et al., 2016). Moreover, vascular hyporeactivity and hyperdynamic circulation result in elevated cardiac index (CI) with low systemic vascular resistance (SVR), which act as confounding factors in the echocardiographic evaluation of these patients. Evaluating the dynamic parameters of fluid responsiveness post-paracentesis could ideally give more reliable results (Simonetto et al., 2019). In a prospective study of patients with ACLF and sepsis-induced hypotension, serial POC echocardiography performed within 6 h of admission demonstrated that the echographic markers of cirrhotic cardiomyopathy (CCM: septal e’ velocity <7 cm/s, E/e’ ratio >15, left atrial volume index >34 mL/m^2^, and tricuspid regurgitant velocity >2.8 m/s) independently predicted vasopressor requirement and 28-day mortality and also increased the discriminative performance of the CLIF-C ACLF and MELD-Na prognostic scores (Kajal et al., 2023). In a prospective study of 372 patients with cirrhosis and acute kidney injury, POCUS-guided volume management at 24, 48, and 72 h supported individualized albumin and fluid administration decisions. In this study, patients were classified as euvolemic, hypovolemic, or hypervolemic based on POCUS evaluation. “Hypovolemia” was defined as patients with left ventricular outflow tract (LVOT) VTI ≤15, IVC diameter <1.3 cm, with IVC collapsibility index (IVCCI) > 40% and A-profile on LUS. “Euvolemia” was defined as LVOT-VTI between 16 and 22, IVC diameter between 1.3 and 2.2 cm, with IVCCI between 20% and 40%, and LUS-A profile. “Hypervolemia” was defined as the LUS changed to a B-profile, with IVC diameter >2 cm and IVCCI <20%. In those who met intermediate criteria, a fluid challenge or passive leg raising (PLR) test was used for volume responsiveness (Premkumar et al., 2025). Lung ultrasound (LUS), performed concurrently, enables the detection of B-lines (>3 per intercostal space in two or more bilateral zones indicating interstitial edema) and consolidations during resuscitation—a clinically relevant concern given the risk of over-resuscitation in this population (Premkumar et al., 2021). The utility of serum lactate in guiding fluid resuscitation in this population is also debated: the hepatic metabolism of lactate is often impaired in patients with cirrhosis, and serum lactate levels should be interpreted with caution in this population (Sterling et al., 2015). Serial lactate measurements are likely more informative, and the trend may correlate better with survival than the absolute values (Funk et al., 2007). The resuscitation fluid of choice in patients with cirrhosis with sepsis is yet to be established. A meta-analysis of different resuscitation fluids in the general population of critically ill patients without cirrhosis reported that balanced crystalloids and albumin decreased mortality more than hydroxyethyl starch and 0.9% saline (Karvellas et al., 2024). Albumin administration is recommended in the management of patients with liver failure with different indications (e.g., large-volume paracentesis, paracentesis-induced circulatory dysfunction, spontaneous bacterial peritonitis, and hepatorenal syndrome), but its use as a resuscitation agent in septic patients with ESLD or ACLF is not well defined (Karvellas et al., 2024). A single-institution RCT comparing 20% albumin with Plasma-Lyte in 100 patients with cirrhosis and sepsis-induced hypotension showed higher rates of shock reversal but no survival benefit and increased pulmonary complications with albumin (Maiwall et al., 2022). Another single-institution trial by Philips et al. (2021) compared 5% albumin with normal saline in 308 hypotensive septic patients with cirrhosis and confirmed that the reversal of hypotension was higher with albumin, with higher 1-week survival (43.5% vs. 38.3%, p = 0.03).

#### Vasoactive agents

Vasopressors should be considered to maintain end-organ perfusion if patients do not respond adequately to fluid resuscitation. A mean arterial pressure (MAP) target of 65 mmHg is recommended in sepsis and critically ill patients, but there are no RCTs that confirm this target in patients with cirrhosis or ACLF; both are populations with a generally lower baseline MAP (Patidar et al., 2020). A recent TARGET-C trial compared strategies targeting either a high- (80–85 mmHg) or low-MAP (60–65 mmHg) in cirrhotic patients with septic shock, demonstrating an improved tolerance of dialysis, lactate clearance, and renal recovery in the high-MAP group, with no survival benefit and a higher incidence of adverse events (Maiwall et al., 2022). Norepinephrine (NE) is recommended as the first-line vasopressor agent to maintain adequate organ perfusion pressure in patients with septic shock (Evans et al., 2021). When adequate MAP is not achieved with NE at doses up to 0.5 μg/kg/min, the addition of vasopressin (0.03 units/min) is recommended by the Surviving Sepsis Campaign Guidelines rather than escalating NE indefinitely; this strategy may reduce NE requirements and catecholamine-related adverse effects (Evans et al., 2021). Terlipressin (a vasopressin analogue with splanchnic vasoconstrictive effects) has an established role in hepatorenal syndrome (HRS-AKI) when combined with albumin (European Association for the Study of the Liver, 2023). A recent RCT compared terlipressin and NE as the first-line vasopressor in ACLF patients with septic shock; terlipressin was shown to be inferior, with a higher mortality rate and a lower number of patients achieving a MAP >65 mmHg at 6 h (Gupta et al., 2025). In refractory shock, the addition of epinephrine as a third vasopressor is recommended. In cardiac dysfunction with persistent hypoperfusion despite adequate volume status and arterial blood pressure, current Survival Sepsis Campaign Guidelines recommend either the addition of dobutamine to norepinephrine or the use of epinephrine alone (Evans et al., 2021). There are no studies in the literature confirming this strategy in patients with cirrhosis or ACLF. Relative adrenal insufficiency is common in patients with ESLD and is associated with higher mortality and complications. Hydrocortisone administration (200 mg/day) is recommended for the treatment of refractory shock requiring high-dose vasopressors (Evans et al., 2021). The few specific studies in patients with ACLF on steroid efficacy in septic shock report a higher rate of shock reversal with steroid treatment (Karvellas et al., 2024; Arabi et al., 2012).

#### Renal replacement therapy

Acute kidney injury is a common finding in sepsis and in ACLF and ESLD with or without sepsis. This population experiences several characteristic types of acute kidney injury: hypovolemic-mediated (prerenal), ischemic/nephrotoxic-mediated (acute-tubular necrosis), and hepatorenal syndrome. Renal replacement therapy (RRT) is frequently required and serves primarily as a support/bridge (managing volume, electrolyte and acid–base derangements, reducing azotemia, and clearing ammonia). Overall, indications for RRT are largely similar between septic patients with ESLD/ACLF and the general population (Pelusio et al., 2024; Evans et al., 2021). In a meta-analysis by Dong et al. (2024), the use of continuous RRT (CRRT) in adult patients admitted to ICU with acute liver failure was associated with overall improved and transplant-free survival.

#### Liver support systems

Many devices have been developed to provide artificial liver support, including albumin dialysis (Molecular Adsorbent Recirculating System (MARS) or single-pass albumin dialysis (SPAD)), plasma separation, and filtration (Prometheus). Despite the theoretical potential of these treatments, results have been inconsistent in terms of transplant-free survival. Therefore, the use of these technologies is still not recommended in patients with ACLF or ESLD, with or without sepsis (Piano et al., 2025). Plasma exchange (PE) has demonstrated promising results in acute liver failure patients, its use resulting in reduced SOFA score, reduced pro-inflammatory cytokines, lactate and ammonia levels, and higher 21-day transplant-free survival (Maiwall et al., 2022). In a meta-analysis that evaluated PE in patients with septic shock, plasma exchange therapy significantly reduced mortality compared to standard medical therapy alone (Hernandez et al., 2024). CytoSorb is an extracorporeal blood purification tool (hemoadsorber) that may be used together with continuous dialysis to remove excessive inflammatory mediators (i.e., cytokines) and to lower elevated bilirubin and myoglobin levels. Brouwer et al. (2019) demonstrated a reduction in 28-day mortality in septic patients treated with CytoSorb (Brouwer et al., 2019). A recent retrospective observational study by Haselwanter et al. (2024) showed that the use of CytoSorb in ACLF patients led to a significant decrease in bilirubin and pro-inflammatory cytokines. While hemoadsorption and PE are emerging as promising therapeutic option, their efficacy and safety await further confirmation to conclusively determine their role in the management of sepsis/septic shock in patients with ESLD/ACLF.

#### Extra-corporeal membrane oxygenation

The use of V-A extra-corporeal membrane oxygenation (ECMO) in refractory septic shock remains a clinical and ethical challenge. Despite technological progress, clear evidence of survival benefit in adults is still lacking. Survival in adults with refractory septic shock supported by V-A ECMO ranges between 15% and 36% (Torre et al., 2025). ELSO registry data suggest a high mortality in unselected cirrhotic patients on ECMO (Rubin et al., 2025). Overall, the quality of available evidence remains limited, and decisions should be individualized by multidisciplinary teams, considering reversibility, bleeding risk, transplant candidacy, and center experience.

### Precision medicine and immune modulation therapies

Sepsis in patients with ESLD and ACLF develops within a background of pronounced systemic inflammation occurring in parallel with a multifactorial immune dysfunction. The pathophysiological mechanisms underlying this state of immune paralysis have been extensively described in the literature and include both innate and adaptive immune abnormalities. This complex immunopathological landscape provides a compelling biological rationale for the investigation of immune-modulating therapeutic strategies in this high-risk population. The concept of precision medicine—matching treatment to an individual patient’s biological response rather than to a syndromic label—has gained substantial momentum in sepsis research. The selection of precision immunotherapy has two proposed steps. In the first step, every patient is measured for a wide panel of biomarkers. Based on the results, the patient is classified as part of a subgroup, or phenotype, based on a certain prevailing mechanism. In the second step, the most appropriate drug is selected based on mechanism classification (Arapis et al., 2025). The PROVIDE clinical trial (Leventogiannis et al., 2022) divided 240 patients with sepsis in three subgroups: 1) macrophage activation-like syndrome (MALS; ferritin >4,420 ng/mL), characterized by excess IL-1 production, intense systemic inflammation, coagulopathy, high short-term mortality (79.1%), and is treated with the IL-1 receptor antagonist anakinra; 2) sepsis-induced immunoparalysis (SII; ferritin ≤4,420 ng/mL, HLA-DR <5,000 receptors/monocyte), characterized by monocyte hypo-responsiveness, susceptibility to secondary infections, 28-day mortality up to 60%, and is treated with recombinant human interferon-γ (rhIFNγ); 3) an intermediate group without extreme immune dysregulation and lower mortality (41,6%). Short-term improvement in organ dysfunction (SOFA score decrease at 7 days) was achieved in 42.9% of the personalized immunotherapy arm vs. placebo (p = 0.042), representing a signal of biological efficacy, though not translated into a 28-day mortality benefit.

In the recent ImmunoSep randomized clinical trial, precision immunotherapy guided by the same classification significantly improved organ dysfunction in 276 patients with sepsis by day 9 compared to placebo (SOFA score decrease in 35.1% vs. 17.9%; *p* = 0.002), without a statistically significant 28-day mortality benefit (Giamarellos-Bourboulis et al., 2025).

Direct application of this framework to ESLD and ACLF faces specific challenges. Serum ferritin is frequently elevated at baseline in liver disease independent of immune activation, reducing its diagnostic specificity for MALS in this population (Bernsmeier et al., 2015). Although anakinra has failed to improve outcomes in unselected septic populations, subgroup analyses have suggested potential benefit in patients with features of macrophage activation syndrome (Opal et al., 1997; Shakoory et al., 2016). Given the central role of systemic inflammation in ACLF, IL-1 blockade represents a biologically plausible therapeutic strategy; however, its role remains under investigation and warrants evaluation in dedicated clinical trials. Monocyte HLA-DR expression may already be reduced in decompensated cirrhosis without sepsis; therefore, there is no validation of biomarker cut-offs (Bernsmeier et al., 2015). Granulocyte colony-stimulating factor (G-CSF) has been explored as a potential immune-restorative therapy in ACLF because of its ability to mobilize hematopoietic stem cells and modulate innate and adaptive immune responses. Early single-center studies suggest an improvement in short-term survival among patients with ACLF treated with G-CSF (Garg et al., 2012; Duan et al., 2013). However, a subsequent large multicenter randomized controlled trial (the GRAFT trial) was prematurely terminated after interim analysis demonstrated no benefit in 90-day transplant-free survival and an increased incidence of severe adverse events in the G-CSF group (Engelmann et al., 2021). Consequently, current EASL Clinical Practice Guidelines on acute-on-chronic liver failure do not recommend the routine use of G-CSF in ACLF (European Association for the Study of the Liver, 2023). Moreover, there is currently no evidence supporting the use of G-CSF in patients with sepsis, underscoring the need for further large, well-designed prospective multicenter trials. Intravenous immunoglobulins (IVIGs) comprise pooled IgG preparations (standard IVIG) or IgM-enriched formulations (IgM-IVIG). Their proposed mechanisms of action include antigen neutralization, Fc-receptor blockade and the modulation of phagocytic cells, attenuation of cytokine responses, interference with complement activation, and broader immunomodulatory effects on innate and adaptive immune cells (Kakoullis et al., 2018). Surviving Sepsis Campaign guidelines advise against the routine use of IVIGs in sepsis and septic shock due to low-quality evidence and uncertain clinical benefit (Evans et al., 2021). To date, no clinical studies have specifically evaluated IVIGs therapy in patients with ESLD or ACLF complicated by sepsis.

### Future directions: PK/PD optimization in cirrhosis

Future research should prioritize pharmacokinetic/pharmacodynamic (PK/PD) optimization strategies tailored to patients with cirrhosis and ACLF, in whom profound pathophysiological alterations significantly affect drug exposure and therapeutic response (Bajaj et al., 2021). Systemic inflammation, capillary leak, expanded volume of distribution, hypoalbuminemia, altered hepatic metabolism, and dynamic renal dysfunction contribute to high interindividual variability and increase the risk of both underdosing and toxicity, particularly for hydrophilic and highly protein-bound antimicrobials. In this context, individualized dosing approaches based on therapeutic drug monitoring (TDM) and model-informed precision dosing (MIPD) represent promising tools to achieve PK/PD targets while limiting the emergence of antimicrobial resistance. The prolonged or continuous infusion of β-lactams, early loading doses, and real-time dose adjustment according to organ function and extracorporeal support should be systematically evaluated in cirrhotic populations. In addition, future studies should integrate population pharmacokinetic models specific to ESLD/ACLF and assess the clinical impact of PK/PD-guided therapy on treatment failure, toxicity, and survival. The implementation of multidisciplinary antimicrobial stewardship programs incorporating clinical pharmacology expertise and TDM may represent a key strategy for improving outcomes in this high-risk population (Guilhaumou et al., 2019; Roberts et al., 2014; Felton et al., 2014).

## Conclusion

Sepsis in end-stage liver disease (ESLD) and acute-on-chronic liver failure (ACLF) is a major determinant of short-term mortality, arising from the interplay between systemic inflammation, cirrhosis-associated immune dysfunction, and early multiorgan failure. Despite advances in diagnostics and supportive care, management still relies primarily on early recognition, prompt antimicrobial therapy, and individualized organ support, while outcomes remain poor, particularly in advanced ACLF and multidrug-resistant infections. Emerging immune-modulating and extracorporeal therapies are biologically plausible but remain investigational, highlighting the need for disease-specific, well-designed clinical trials to improve prognosis in this high-risk population.
